# How often do cancer researchers make their data and code available and what factors are associated with sharing?

**DOI:** 10.1186/s12916-022-02644-2

**Published:** 2022-11-09

**Authors:** Daniel G. Hamilton, Matthew J. Page, Sue Finch, Sarah Everitt, Fiona Fidler

**Affiliations:** 1 MetaMelb Research Group, School of BioSciences, University of Melbourne, Melbourne, Australia; 2 Melbourne Medical School, Faculty of Medicine, Dentistry & Health Sciences, University of Melbourne, Melbourne, Australia; 3 School of Public Health & Preventive Medicine, Monash University, Melbourne, Australia; 4 Melbourne Statistical Consulting Platform, School of Mathematics and Statistics, University of Melbourne, Melbourne, Australia; 5 Sir Peter MacCallum Department of Oncology, University of Melbourne, Melbourne, Australia; 6 School of Historical and Philosophical Studies, University of Melbourne, Melbourne, Australia

**Keywords:** Data sharing, Code sharing, Oncology, Cancer, FAIR principles

## Abstract

**Background:**

Various stakeholders are calling for increased availability of data and code from cancer research. However, it is unclear how commonly these products are shared, and what factors are associated with sharing. Our objective was to evaluate how frequently oncology researchers make data and code available and explore factors associated with sharing.

**Methods:**

A cross-sectional analysis of a random sample of 306 cancer-related articles indexed in PubMed in 2019 which studied research subjects with a cancer diagnosis was performed. All articles were independently screened for eligibility by two authors. Outcomes of interest included the prevalence of affirmative sharing declarations and the rate with which declarations connected to data complying with key FAIR principles (e.g. posted to a recognised repository, assigned an identifier, data license outlined, non-proprietary formatting). We also investigated associations between sharing rates and several journal characteristics (e.g. sharing policies, publication models), study characteristics (e.g. cancer rarity, study design), open science practices (e.g. pre-registration, pre-printing) and subsequent citation rates between 2020 and 2021.

**Results:**

One in five studies declared data were publicly available (59/306, 19%, 95% CI: 15–24%). However, when data availability was investigated this percentage dropped to 16% (49/306, 95% CI: 12–20%), and then to less than 1% (1/306, 95% CI: 0–2%) when data were checked for compliance with key FAIR principles. While only 4% of articles that used inferential statistics reported code to be available (10/274, 95% CI: 2–6%), the odds of reporting code to be available were 5.6 times higher for researchers who shared data. Compliance with mandatory data and code sharing policies was observed in 48% (14/29) and 0% (0/6) of articles, respectively. However, 88% of articles (45/51) included data availability statements when required. Policies that encouraged data sharing did not appear to be any more effective than not having a policy at all. The only factors associated with higher rates of data sharing were studying rare cancers and using publicly available data to complement original research.

**Conclusions:**

Data and code sharing in oncology occurs infrequently, and at a lower rate than would be expected given the prevalence of mandatory sharing policies. There is also a large gap between those declaring data to be available, and those archiving data in a way that facilitates its reuse. We encourage journals to actively check compliance with sharing policies, and researchers consult community-accepted guidelines when archiving the products of their research.

**Supplementary Information:**

The online version contains supplementary material available at 10.1186/s12916-022-02644-2.

## Background

The notion that scientific findings should be independently verifiable is a key tenet of science, with this principle famously being enshrined in 1660 in the Royal Society of London’s motto “Nullius in verba” or “Take nobody’s word for it”. However, the extent to which researchers adhere to this value in practice varies across fields. In the context of contemporary cancer research, the routine reuse of previously collected data to verify important findings, validate developed models, generate evidence to guide clinical decision-making and identify new avenues for research, clearly signifies that the cancer research community considers open data a valuable commodity.

For example, databases like The Cancer Genome Atlas (TCGA), which currently curates more than 2.5 petabytes of data collected from over 11,000 cancer patients spanning 33 tumour types (including 10 rare cancers), has been used by thousands of cancer researchers since the initiative was launched in 2005 [1]. Similarly, more than 17,000 articles using cancer incidence, prevalence and survival data from the Surveillance, Epidemiology, and End Results (SEER) Program of the US National Cancer Institute (NCI) for the primary analysis were published between 1973 and 2020 [2]. We also note that a third of all individual participant data meta-analyses (IPDMA) published between 1987 and 2015 were conducted by cancer researchers, and that IPDMAs also accounted for almost 10% of reviews cited by National Comprehensive Cancer Network (NCCN) clinical practice guidelines in 2019 [3, 4]. However, despite regular reuse of previously collected data by medical researchers for primary and secondary research purposes, several barriers such as the as navigation of intellectual property and privacy issues, loss of academic productivity, and time and resource burdens appear to be disincentivising researchers from sharing their own original data [5, 6].

In spite of these concerns, a growing number of cancer research stakeholders are calling for increased availability of data and code from cancer research. For example, funders of cancer research continue to strengthen their policies on data availability, with some like the National Institutes of Health (NIH) already mandating sharing of data under certain circumstances [7]. Equally, a growing minority of journals require authors to include a data availability statement and publicly share data and code as a condition of publication [8]. Some of these journals (e.g. *Nature Cancer*) also review code and software deemed integral to submitted research and use them to computationally reproduce reported findings. The latter occurrence represents another useful service journals could provide to protect the cancer research community against the publication of erroneous research, particularly when appreciating that errors in data and analyses contributed to the retraction of more than a thousand medical papers between 2017 and 2021 [9].

We also note very high levels of support from medical journal editors of requests from reviewers to access manuscripts’ raw data [8]. Other research stakeholders, such as the rare diseases communities, are also calling for greater availability of data to increase opportunities to re-analyse historical data to answer new research questions (i.e. secondary analyses) and combine historical data together to enhance our understanding of old ones (i.e. IPDMAs) [10, 11]. In the context of cancer research, both these types of research designs have been instrumental in shaping our understanding of topics like PSA-based screening for prostate cancer and overcoming low statistical power to reveal the benefits of treatments such as adjuvant tamoxifen for breast cancer [12, 13].

While significant progress has been made towards increasing the availability of the products of research (i.e. data, code and materials), previous research on the topic has reported low availability of data (0–6%) and code (0–2%) across many medical fields between 2014 and 2018 and beyond [14–27]. Similar observations have also been made in oncology, with one study by Walter and colleagues [21] observing that only 2% and 0% of 45 sampled cancer publications in 2014 shared sufficient data and code to computationally verify all reported findings; with this rate not increasing over the subsequent three year period. Furthermore, other research in medicine has shown sub-optimal compliance with journal data and code sharing policies [28–30]. In the current exploratory study, we build on previous research to investigate how frequently cancer researchers share the data underlying their research, as well as the code used to perform statistical analyses in a large random sample of published cancer studies. We investigate the level of compliance with journal policies, as well as compliance with guidelines that ensure outputs are available in a way that maximally facilitates their reuse (i.e. FAIR principles [31]) — a consideration which to the authors’ knowledge has only been investigated by a single previous study in medicine [32]. Finally, we explore the link between some novel factors and data availability, such as the rarity of the cancer studied, the use of publicly available data in the research project and the posting of pre-prints. We also explore factors such as the collection of data from human research subjects, open access publication models, and subsequent citation rates, which have been associated with data sharing and withholding in multiple fields in the past [33, 34].

## Methods

### Study design

The following study is a cross-sectional analysis of cancer-related articles published between January 1st and December 31st, 2019. The full study protocol outlining the methods of the study was publicly registered on the Open Science Framework (OSF) on March 18th, 2020, prior to running the literature search [35]. Important aspects of the methods are described briefly below. As the research subjects of interest were scientific publications, ethics approval was not required.

### Article selection

PubMed was searched on March 18th, 2020, to locate oncology-related publications indexed in MEDLINE and PubMed Central in 2019. The search results were randomised in R using the sample function, then titles and abstracts, followed by full-text articles, were independently screened in Google Sheets by two coders in parallel (DGH; JM) until the required number of eligible studies (*N*=306) were identified. Any discrepancies between the two coders were resolved via discussion, or adjudication by another member (MJP). The eligibility criteria used for the study were as follows: (1) the article presents results of a study which recruited, involved or concerned populations, cell lines, animal analogues and/or human participants with any cancer diagnosis (benign or malignant); (2) the article was not a case report, conference abstract, synthesis of existing research (e.g. guideline, review or meta-analysis) or an opinion/news piece (e.g. editorial, letter, non-systematic expert review), (3) the article was not retracted, flagged as a duplicate publication, issued with an expression of concern, or any other reasons that would undermine trust in the research, and (4) the article was written in English, available in full-text and published (electronically or in-print) between January 1st and December 31st, 2019. The full search strategy, search records, and screening results are freely available on the project’s OSF page [35].

### Study outcomes

The primary outcomes of interest included the public sharing of data and code. For the purposes of the study, we defined ‘code’ as the step-by-step syntax outlining all commands used within a statistical software to execute any reported analyses, and ‘data’ as quantitative (countable) or qualitative (textual, visual or audio) sample-level information required to reproduce and verify any or all reported analyses (including data visualisations). For example, quantitative patient-level data that would theoretically enable an independent researcher to recalculate and verify the median age of a reported cohort. In addition to the above definition of data, we also considered data sharing in the context of macromolecular structures, nucleic acid and protein sequences, microarray data and Nuclear Magnetic Resonance spectroscopy data (e.g. sharing of free induction decays [36]). However, the preparation, deposition and availability of specimens and other research materials (e.g. reagents), as well as other forms of data that did not meet the above definition were considered out of scope.

Two specific types of ‘sharing’ were evaluated as part of this study. The first was data and code sharing according to author declarations alone (‘reported availability’). This was defined as the presence of text, occurring anywhere in the article (e.g. in the Methods section, within a formalised data/code availability statement) or supplementary material, that explicitly stated that some or all data or code underpinning the results were available, and where it can be accessed. We did not regard statements such as ‘supplementary data are available’ or ‘data or code are available on request’ as declarations of availability. Nor did we deem references to publicly available datasets used to complement original research (e.g. to validate models) as data sharing. In the context of research that only used publicly available data (e.g. SEER data), authors needed to provide detailed information on how (or whether) the specific dataset(s) used to generate the results of the study (as opposed to the most recent iteration) could be accessed.

Secondly, data reported as available were further investigated to see determine the level of compliance with the FAIR Data guiding principles [31] via an abbreviated version of the Australian Research Data Commons’ FAIR data self-assessment tool [37]. Specifically, for the purposes of this study, data were considered FAIR-compliant if they were (1) assigned both a unique and permanent identifier, (2) posted to a general, domain-specific or local institutional registry listed on re3data.org, (3) freely accessible, or accessible to researchers under explicitly stated conditions, (4) archived in a non-proprietary format (e.g. .csv, .tsv, .txt) and (5) associated with a license outlining its terms of use. Items 1-2, 3, 4 and 5 relate to the ‘Findable’, ‘Accessible’, ‘Interoperable’ and ‘Reusable’ principles respectively. If two or more datasets were posted, all were assessed and the dataset with the highest compliance was reported.

Numerous other variables were also of interest to the study, with some key variables including (1) open access status with categories defined as per Piwowar and colleagues [38]; (2) the cancer research area classified according to the International Cancer Research Partnership’s (ICRP) ‘Common Scientific Outline’ (CSO) classification system; (3) whether the study investigated cancers classified as rare by the RARECARE project (i.e. cancers with incidence rates of less than 6 in 100,000); and (4) the number of citations accrued by the first and second year post-publication as per OpenCitation’s COCI database [39] (this was a deviation from our study protocol). Journal websites were also manually checked between April 28th and 29th, 2020, for policies governing data and code sharing, as well as the addition of availability statements. Policies were coded into the following categories: journal requires all empirical articles to share data (‘all mandatory’), journal requires some articles to share data but not others (e.g. clinical trials, research using x-ray crystallography) (‘some mandatory’), journal requires authors to share data in response to reasonable requests (‘share on request’), journal encourages sharing of data (‘encourage’) and journal does not have a policy on data sharing (‘no policy’). A comprehensive list of all the outcomes of interest to the study and their definitions are available in the study protocol and in the data dictionary on the OSF project page [35].

### Data extraction

A pre-defined Google Form for data extraction was created and piloted prior to use. Primary and secondary outcome data were extracted by two authors independently in parallel (DGH; JM) for the first 198 articles (65%), with differences between coders resolved by consensus or a third party (MJP). Kappa coefficients and average percentage agreements were then calculated for each of the seven primary and secondary outcome measures, following which a single author (DGH) extracted outcome data for the remaining 108 articles when inter-coder reliability was determined to be sufficiently high for the first 198 articles (kappa coefficient greater than 0.70 and the percentage agreement greater than 95% for each domain). Refer to the OSF project page for the results of the reliability analysis [35].

### Statistical considerations

In recognition of previous research in biomedicine that reported 13% of articles between 2015 and 2017 both discussed and shared a functional link to research data [40], and assuming a slightly higher estimate for the oncology literature of 15%, a random sample of 306 articles was chosen to ensure a 95% credible interval (CI) width less than 8% using the modified Jeffrey’s Interval proposed by Brown et al. (2001) [41]. The method proposed is a Bayesian approach to interval estimation of binomial proportions which has been shown to provide good nominal coverage, particularly as sample proportions approach 0 (or 1) [41]. The approach uses the non-informative Jeffrey’s conjugate prior (i.e. beta distribution with parameters 0.5 and 0.5) and results in a posterior distribution of beta (*x* + 0.5, *n* − *x* + 0.5), where *x* is the number of successes and *n* the number of Bernoulli trials.

All categorical data are presented as counts and proportions. Continuous data are presented as means and standard deviations or as medians and interquartile ranges when data were highly skewed. Credible intervals around sample proportions for binary variables using the modified Brown method were calculated using the DescTools package [42]. In a further analysis, simple and multiple logistic regression models were also generated using the glm function in R to estimate unadjusted and adjusted odds ratios and 95% confidence intervals for data and code sharing predictors while controlling for possible confounding effects of the categorical journal data sharing policy variable (no policy, encourage, some mandatory and all mandatory). All statistical analyses were performed in R (v4.2.0). All data needed to reproduce the reported analyses have been made publicly available on the Open Science Framework [43].

## Results

### Characteristics of included studies

The PubMed search was performed on March 18th, 2020, and yielded 200,699 records. Search results were randomised, then titles and abstracts, then full-text articles, were screened until the required 306 eligible articles were identified. Key characteristics of the 306 included studies (published in 235 unique journals) are reported in Table 1 (refer to Additional file 1: Table 1 for more detailed cross-tabulations).

**Table 1:** Characteristics of the included studies (*N*=306)

	*N*	%
2018 Journal Impact Factor		
No JIF	27	9%
0–5	200	65%
5–10	63	21%
10+	16	5%
Open access		
No	155	51%
Yes	151	49%
Journal data sharing policy		
No policy	67	22%
Encourage	147	48%
Share on request	4	1%
Some mandatory	59	19%
All mandatory	29	9%
Journal code sharing policy		
No policy	202	66%
Encourage	88	29%
Share on request	6	2%
Some mandatory	3	1%
All mandatory	7	2%
Journal DAS policy		
Not required	255	83%
Required	51	17%
Location of first author		
North America	72	24%
Asia	139	45%
Europe	72	24%
North Africa/Middle East	11	4%
Oceania	5	2%
South America	2	1%
Central America/Caribbean	2	1%
Sub-Saharan Africa	3	1%
Research area (CSO classification)^a^		
Biology	69	23%
Aetiology	20	7%
Prevention	4	1%
Detection, diagnosis and prognosis	92	30%
Treatment	112	37%
Control, survivorship and outcomes	51	17%
Clinical trial		
No	291	95%
Yes (phase III)	4	1%
Yes (other phase)	11	4%
Cancer rarity		
Common	150	49%
Rare	118	39%
Mixed	28	9%
Other	10	3%
Data sourced from^a^		
Human subjects	177	58%
Human cells	111	36%
Animal analogues	66	22%
Animal cells	65	21%
Commercial cell lines	116	38%
Simulations	3	1%
Collected data from human participants		
Yes	225	74%
No	81	26%
Used publicly available data		
No	218	71%
Yes (partially)	57	19%
Yes (exclusively)	31	10%
Citations accumulated		
Median (IQR) (year 1)	2	0 to 4
Median (IQR) (year 2)	3	1 to 7

^a^Percentages do not add up to 100% due to multiple answers being possible

There was a median of eight authors per article (IQR: 6-11), with 92% of the first authors being affiliated with institutions in Asia (139, 45%), North America and Europe (both 72, 24%). Most articles investigated new or existing cancer treatments (112, 37%), detection, diagnostic and prognostic methods (92, 30%) or biological processes (69, 23%). Of the 306 eligible studies, 81 (26%) collected and analysed data derived only from non-human participants. The most studied cancers included breast cancer (33, 11%), bowel cancer (25, 8%), lung cancer (24, 8%), brain cancer (23, 8%), or a combination of multiple cancers (48, 16%). Almost half of the eligible articles investigated rare cancers either in isolation (118, 39%), or in combination with other common variants (28, 9%). Only 15 articles (5%) reported the results of clinical trials, four of these reporting the findings of randomised controlled trials.

Most articles were published in subscription and gold open access journals (51% and 31% respectively), and in journals with a 2018 Impact Factor less than five (200, 65%). Almost a third of articles were subject to journal data sharing policies that either: required authors to share all data associated with the research (29, 9%), or some data under certain circumstances (59, 19%). Almost one in five articles (51, 17%) were also required by the submitting journal to complete a data availability statement. In contrast to data sharing policies, mandatory code sharing policies were much less common (10, 3%).

Other statements designed to improve transparency, such as statements outlining whether authors had any competing interests, or whether the study received funding were common (91% and 81% respectively). Similarly, most studies also declared whether ethics approval was obtained or not required (76%). In contrast, 65 (21%) and 8 (3%) articles featured formalised, stand-alone sections dedicated to addressing data and code availability respectively (i.e. data and code availability statements). Declarations of the use of open science practices, such as the public sharing of research protocols and study pre-registration were rare (2% and 4% respectively) — the latter being almost exclusively practised by clinical trialists (11/12, 92%).

### Availability of raw data

Of the 306 studies assessed, 59 declared that some or all data were publicly available (19%, 95% CI: 15–24%), 39 declared that data were available upon request (13%, 95% CI: 9–17%) and the remaining 208 articles stated that data were not available (*N*=3) or did not provide any information on availability (*N*=205) (68%, 95% CI: 63–73%). Of the 59 affirmatory declarations, 27 (46%) were located in dedicated data availability statements, with the remaining 32 (54%) declarations being located in other parts of the manuscript (e.g. the Methods section, supplementary material). Excerpts of all 59 affirmative declarations are available on the OSF [35].

**Fig. 1:**
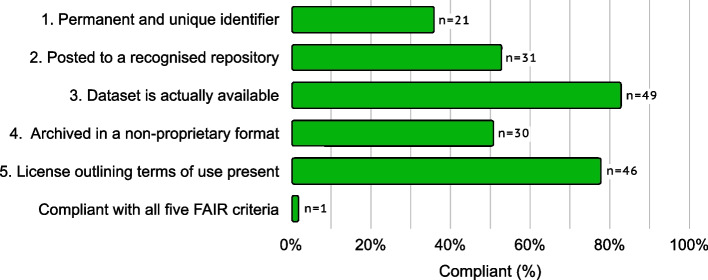
FAIR data assessment of the 59 studies that declared that data were publicly available

When the 59 studies that declared data were publicly available were investigated with respect to their compliance with the FAIR principles, 49 (83%) were observed to have deposited data in a freely (or theoretically) accessible location, most commonly into data repositories (31/49, 63%) or as supplementary material on the journal website (10/49, 20%). Furthermore, when data were available for assessment, only one study (0.3%, 95% CI: 0–2%) was found to comply with the remaining four FAIR assessment criteria (Fig. 1). The most common reasons for non-compliance included the lack of both a unique and permanent identifier (38/59, 64%), archival in a proprietary format (29/59, 49%) and not depositing data in a recognised repository (28/59, 47%).

### Statistical considerations and code sharing

Of the 306 eligible articles, 274 reported inferential statistics (90%, 95% CI: 86–93%). Of these 274 studies, ten reported that some or all code was publicly available (4%, 95% CI: 2–6%), two reported that code is available on request (1%, 95% CI: 0–2%) and 262 did not provide any information on code availability (96%, 95% CI: 93–98%). Of the ten declarations, seven originated from data or code availability statements, and the remaining three appeared in other parts of the manuscript.

Of the 274 studies that reported inferential statistics, 255 (93%, 95% CI: 90–96%) did not report performing formalised sample size calculations prior to collecting data. Furthermore, a quarter of the studies that used inferential statistics also did not report which statistical analysis software they used to analyse their data (70/274, 25%). When reported, the most frequently used software, alone or combination with others, included: SPSS (*N*=92), GraphPad (*N*=58), R (*N*=37), SAS (*N*=19) and Stata (*N*=12).

### Compliance with journal policies

Less than half of the 29 articles that were subject to a blanket mandatory data sharing policy were observed to make data available (14/29, 48%, 95% CI: 31–66%). Furthermore, of the six studies that performed inferential statistics and were subjected to a blanket mandatory code sharing policy, none reported sharing code. In contrast, 88% of articles (45/51, 95% CI: 77–95%) that were required to complete a data availability statement complied.

When comparing the effectiveness of data sharing policies, authors publishing work in journals with mandatory data sharing policies were associated with nearly a tenfold increase in the odds of sharing data than those publishing in journals with no policy (OR: 9.5, 95% CI: 3.26–30.85). This association was also observed for articles published in journals that require authors to share under some circumstances but not others (OR: 3.5, 95% CI: 1.30–10.37). In contrast, authors that submitted to journals that encouraged data sharing appeared to be no more likely to share data than authors publishing in journals without a data sharing policy (OR: 1.1, 95% CI: 0.41–3.14). Interestingly, the odds of sharing data were more than twice as high for authors that were required to complete a data availability statement in comparison to authors who were not (OR: 2.4, 95% CI: 1.14–4.78). However, this relationship was not found to be statistically significant when the effect of journal data sharing policies was accounted for.

### Predictors of data and code sharing

**Fig. 2:**
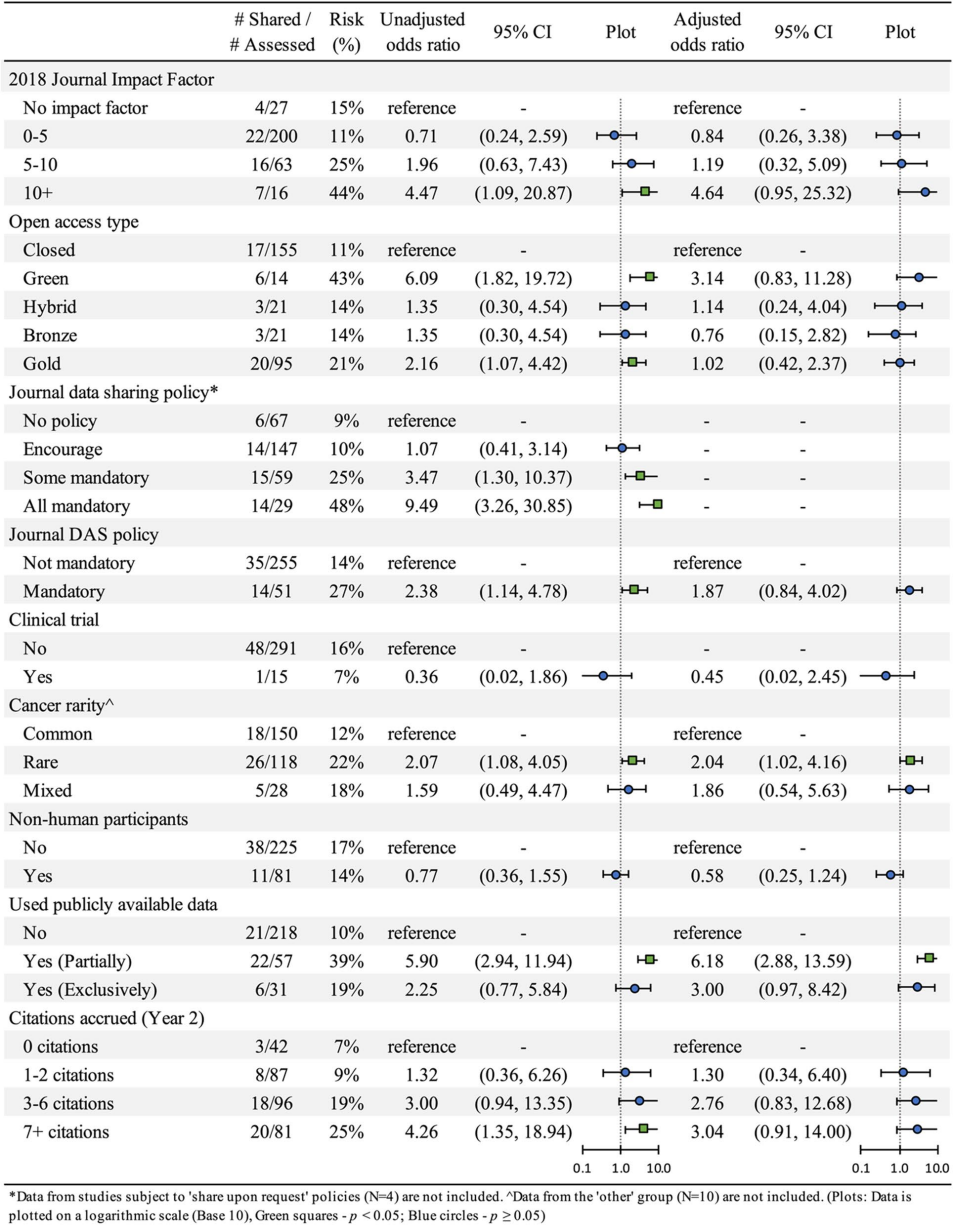
Unadjusted odds ratios, adjusted odds ratios (controlling for data sharing policy) and 95% confidence intervals for the association between study characteristics and data availability

The association between sharing and withholding data with several journal and article characteristics are reported in Fig. 2. Publishing in a journal with 2018 Journal Impact Factor less than ten was not associated with greater rates of sharing in comparison with journals not indexed in Clarivate Analytic’s Web of Science. In contrast, the odds of sharing data were 4.5 times (95% CI: 1.09–20.87) higher for researchers who published in a journal with an Impact Factor greater than ten compared to those who published in a journal with no impact factor. Articles in the upper quartile for citations accrued within two years were also associated with higher odds of data sharing than those in the bottom quartile (OR: 4.3, 95% CI: 1.35–18.94). However, both relationships were not found to be statistically significant when the effect of journal data sharing policies was accounted for.

The odds of sharing data were estimated to be 2.2 (95% CI: 1.07–4.42) and 6.1 times (95% CI: 1.82–19.72) higher for researchers that published in gold open access journals, or publicly shared a pre-print version of their paper both in comparison to researchers whose articles were paywalled. However, the odds of both effects halved in size when journal policies were controlled for.

We noted a 42% reduction (95% CI: 0.25–1.24) in the adjusted odds of sharing data among researchers who only studied non-human participants, compared to those who included data derived from human participants. Similarly, we noted a 55% decrease (95% CI: 0.02–2.45) in the odds of sharing data among researchers publishing the results of clinical trials in comparison to researchers presenting the results of other study designs, even after controlling for the effects of data sharing policies. However, the low precision limits our ability to interpret both results conclusively. In contrast, the odds that a researcher studying rare cancers or using public data to supplement their original findings shared their data were more than twice (OR: 2.1, 95% CI: 1.08–4.05) and almost six times (OR: 5.9, 95% CI: 2.94–11.94) higher respectively than those who did not. The strength and significance of both associations remained stable even after controlling for the effects of data sharing policy.

Like the data sharing predictors, we noted more than a fivefold increase in the odds of reporting code to be available for researchers who shared data than for those that did not share data (OR: 5.6, 95% CI: 1.50–21.00). Additionally, the odds of reporting code to be available were 11.8 times (95% CI: 1.32–106.72) greater for researchers who publicly shared a pre-print version of their paper in comparison to researchers that published in subscription journals.

## Discussion

The current study has aimed to provide an accurate estimate of the frequency with which oncology researchers declared research data were available in 2019, as well as determine how often such declarations linked to the stated products. We note that one in five articles (59/306) reported that some data underpinning the research were available and that the majority of these declarations (83%) did link to data; however only one did so in a way that conformed with key best practice archiving guidelines. The current study also explored the association between data availability and a series of factors and noted that the odds of sharing data were two- and sixfold higher among researchers studying rare cancers and using publicly available data to complement original research respectively — even after controlling for the effect of journal data sharing policies. However, we did not find any evidence to suggest that triallists or researchers studying non-human participants were more likely to share. We also note that authors who shared data and shared a pre-print version of their paper were much more likely to report code to be available as well.

Increasing concerns about the reliability of scientific claims continue to fuel research into the reproducibility, robustness, and generalisability of scientific findings. In modern medical research, such concerns have sparked several influential research initiatives in pre-clinical medicine and cancer biology which have greatly reshaped our understanding of the extent and causes of irreproducible research [44–48] — an issue which is of particular interest to the medical research industry given the high failure rate of clinical trials and the increasing costs of drug development and demand for more effective treatments [45, 49].

One key obstacle to reproducible research that has been highlighted by this body of research includes the overall low public availability of data, code, and materials. For example, a recent initiative by Errington and colleagues [50] which was only able to successfully complete replications for a quarter of the shortlisted cancer biology experiments cited low public availability of data (4/193, 2%) and code (1/78, 1%) as a major impediment (i.e. a key barrier to computing effect sizes, performing power analyses, identifying the statistical analysis strategy).

The observation of the low availability of data and code in cancer research, as well as medical research more broadly, is not new. Rather our observations that 19% and 4% of cancer researchers declared data and code were publicly available are consistent with several previous studies reporting low, but increasing, declaration rates ranging between 3–24% and 0–2% respectively across a number of other medical fields between 2014 and 2018 [14–24], as well as more recent estimates [25–27]. However, while data and code sharing declarations were similarly uncommon in our study, encouragingly, we note rates were three to four times higher than a study by Walters and colleagues [21] who observed that 6% and 0% cancer researchers declared and data and code were respectively available between 2014 and 2018 across a random sample of 194 oncology articles.

The increase of declarations over time — particularly data availability declarations — is likely due to the growing number of medical journals that are adopting stronger policies on data and code sharing, particularly those that are requiring the addition of availability statements. For example, we note that a quarter of the unique 235 journals analysed in our study had adopted a mandatory data sharing policy for some or all data, which is higher than a previous survey of medical journal editors in the previous year [8]. Furthermore, the proportion of articles that included a formalised data availability statement in our study (65, 21%), which is also now a requirement for articles reporting the results of clinical trials [51], is also consistent with prior research in medicine such as Wallach and colleagues [40] who observed a substantial rise in the proportion of biomedical articles including an availability statement from 0% in 2009, up to 25% in 2017.

While progress is clearly being made on increasing transparency surrounding whether data is available or not, we note a large discrepancy between affirmative declarations and the sharing of data in a way that facilitates its reuse. Specifically, we noted that only one of the 59 articles that declared data was available complied with our FAIR assessment. This observation, depending on how availability for reuse is defined, is unfortunately consistent with this body of research which has reported 50–100% reductions in availability following interrogation of sharing statements [14–24]; with factors such as the lack of unique and permanent identifiers, meta-data and licensing terms being noted as major pitfalls [32, 52]. Furthermore, while we also noted a strong relationship between mandatory data sharing policies and actual data availability, we unfortunately also observed similarly sub-optimal compliance with these policies too; a finding that has been noted by other studies both inside and outside of medicine [28, 53, 54]. However, compliance issues aside, it is important to note that such policies are likely much more effective at prompting sharing than other strategies such as ‘share on request’ policies which have been associated with varying compliance rates between 4 and 35% [29, 55–57], as well as encourage policies [53] or no policy at all [55].

In contrast to the growth of data sharing declarations over time, despite claims that code sharing is becoming increasingly normalised across many scientific fields [58], we note persistently low code sharing rates in oncology and medicine more broadly since 2014 [14–26]. Furthermore, none of the six studies in our sample that were subject to mandatory code sharing policies reported code to be available. A finding which is consistent with the only other study to the authors’ knowledge that has examined compliance with code sharing policies in medicine by Grayling and Wheeler [30] who reported that only 18% of the 91 methodological articles describing novel adaptive clinical trial designs that were subject to mandatory sharing policies made their code available. However, interestingly all six studies (which were also subject to mandatory data sharing policies) did address data availability, which may suggest that cancer researchers are less aware of code sharing policies than data sharing policies. An outcome that has been documented previously in a small survey by Christian and colleagues in 2020 [59]. Low compliance could also be explained by a lack of familiarity with what code sharing entails, even within the medical methodological research community. However, it cannot be explained by the inability to generate code given more than 90% of studies examined in both our study and that by Grayling and Wheeler [30] used software that are all syntax-based programs (SPSS, R, SAS, Stata), or allow users to generate files that preserve the decisions made when analysing data (GraphPad).

The findings of the current study raise some important implications and recommendations for various cancer research stakeholders. Firstly, for the publishers of cancer research, based on our findings it is likely that a substantial number of cancer researchers are not complying with mandatory data and code sharing policies. Consequently, we recommend that journals that have, or are considering, implementation of stringent data and code sharing policies, also ensure that they incorporate mechanisms into their editorial workflows to ensure submitting authors comply with such policies. Secondly, while data sharing rates appear to have increased relative to previous estimates in cancer research [21], we note suboptimal compliance with data management and stewardship guidelines like the FAIR principles [31]. As a consequence, publicly shared research products may not be as discoverable and ultimately reusable as the data creators may have envisaged. We recommend that cancer researchers planning to publicly archive research data and code consult resources like re3data.org to find the most appropriate repository for their needs, include detailed meta-data alongside posted datasets, and where applicable, pay particular care to ensure that the terms of use of the data are clearly reported to maximise other researchers’ ability to reuse deposited assets. Furthermore, we strongly encourage that all researchers routinely provide as much clarity as possible on the conditions governing access and reuse of their research data and code (even if access to such products is restricted), as well as reflect on, and continuously assess their own archiving practices to ensure the products of their research are preserved for potential reuse in the future – whether that is only by the original researchers themselves, or by other members of the cancer research community. Lastly, we also recommend that meta-researchers planning to investigate data sharing and factors influencing data sharing behaviours in the future connect with cancer research stakeholders to explore barriers to sharing, empirically test strategies for how sharing rates might be improved, as well as consider research into underlying theoretical frameworks, particularly institutional and individual factors [60, 61] (e.g. theory of planned behaviour, institutional theory) when designing hypothesis-driven research.

We note some strengths of our study. First, our sample size was 4–10 times bigger than the annual estimates of previous research evaluating data and code sharing rates in both oncology and medicine more broadly between 2014 and 2018 [14–24]. Random sampling of articles also allowed us to make inferences about sharing rates more broadly than studies focused on sharing rates for articles published in specific journals (e.g. high-impact journals), or using certain study designs (e.g. randomised controlled trials). Second, we manually examined entire articles and supplementary materials for declarations of data and code sharing. Third, our study is one of the very few studies to assess other factors associated with best practices in archiving and sharing, such as data licensing, formatting, and discoverability, as well as assess sharing rates in the setting of rare cancers. However, we also recognise a few limitations. First, while journal policies on data and code sharing were captured shortly after running the literature search, there was still a 4- to 14-month delay between the publication of articles and the collection of this policy information. Consequently, there is a chance that some articles may have been subject to different availability policies that were superseded during this period. Second, the study has only focussed on sharing rates within a single year and so cannot make any commentary about how sharing rates may have changed over time. Third, not all data were extracted in duplicate by two authors and due to the use of simple random sampling of articles, we note underrepresentation of some important classes (e.g. clinical trial design, high-impact papers, pre-printed publications) in our analyses which have resulted in low precision. Lastly, it is also likely that sharing rates would have further increased since 2019. A notion that is supported by a recent and large study which observed an increase in data sharing declarations among papers available in PubMed Central between 2019 and 2020 [25]. Sharing in the modern-day context may also have been further enhanced beyond some of these estimates in the wake of global COVID-19 pandemic. Particularly as journals take on stronger positions on data availability, and as the uptake of other open science practices such as pre-printing have substantially increased [62, 63]. Both these propositions are key questions of an ongoing individual participant data meta-analysis [64].

## Conclusions

Despite some progress being made towards increasing the availability of the products of research, data and code sharing in oncology has been observed to occur infrequently, and at a rate lower than would be expected given the prevalence of mandatory sharing policies. There is also a large gap between the number of cancer researchers declaring data to be available and those archiving it in a way that maximally facilitates its reuse. Both journal editors and reviewers can help with this through more active enforcement of mandatory data and code sharing policies. Given the extent to which the cancer research community already regularly reuses data to verify important findings, validate developed models, generate evidence to guide clinical decision-making and identify new avenues for research, we also strongly encourage that authors provide as much clarity as possible on the conditions governing access and reuse of their research data and code, even if access to such products is restricted. Additionally, we recommend that cancer researchers and institutions consult relevant community-accepted guidelines like the FAIR principles when archiving the products of their research to maximise their value for potential reuse in the future — whether that is only by the original researchers themselves, or by other members of the cancer research community.

## Supplementary Information

**Additional file 1: Table 1.** Characteristics of included studies by sharing type (*N*=306).

## Data Availability

The data, materials and code supporting the conclusions of this article are publicly available on the Open Science Framework (DOI: 10.17605/OSF.IO/Z3BFT).
