# Supplementary material for: How often do cancer researchers make their data and code available and what factors are associated with sharing?
Source: BMC Med. 2022 Nov 9;20:438. doi: 10.1186/s12916-022-02644-2 (PMC9646258; doi:10.1186/s12916-022-02644-2)
Supplement: Supplementary file 1 — Additional file 1: Table 1. Characteristics of included studies by sharing type (N=306). [file 12916_2022_2644_MOESM1_ESM.pdf]

Additional File 1: Table 1. Characteristics of included studies by sharing type (N=306).

	All articles (N=306)		Actual data sharing				Reported data sharing					
			Data publicly available (N=49)		Not available (N=257)		Data publicly available (N=59)		Available on request (N=39)		Not available (N=208)	
	N	(%)	N	(%)	N	(%)	N	(%)	N	(%)	N	(%)
Publisher												
Elsevier BV	65	21%	9	18%	56	22%	9	15%	4	10%	52	25%
Springer	62	20%	12	24%	50	19%	15	25%	12	31%	35	17%
Wiley	39	13%	2	4%	37	14%	2	3%	8	21%	29	14%
MDPI AG	13	4%	6	12%	7	3%	6	10%	0	0%	7	3%
Informa UK Limited	12	4%	1	2%	11	4%	1	2%	2	5%	9	4%
Wolters Kluwer	9	3%	1	2%	8	3%	1	2%	0	0%	8	4%
American Association for Cancer Research	8	3%	2	4%	6	2%	2	3%	0	0%	6	3%
Frontiers Media SA	7	2%	2	4%	5	2%	4	7%	2	5%	1	0%
Spandidos Publications	6	2%	0	0%	6	2%	2	3%	4	10%	0	0%
American Chemical Society	5	2%	0	0%	5	2%	1	2%	0	0%	4	2%
Mary Ann Liebert Inc	5	2%	1	2%	4	2%	1	2%	0	0%	4	2%
Oxford University Press	5	2%	2	4%	3	1%	2	3%	0	0%	3	1%
SAGE Publications	5	2%	2	4%	3	1%	2	3%	0	0%	3	1%
Other	65	21%	9	18%	56	22%	11	19%	7	18%	47	23%
Date published												
Q1 2019	62	20%	9	18%	53	21%	11	19%	4	10%	47	23%
Q2 2019	58	19%	10	20%	48	19%	11	19%	5	13%	42	20%
Q3 2019	75	25%	11	22%	64	25%	14	24%	8	21%	53	25%
Q4 2019	111	36%	19	39%	92	36%	23	39%	22	56%	66	32%
Number of authors												
Median (IQR)	8	6 to 11	9	6 to 15	8	5 to 10	9	6 to 14	8	6 to 12	7	5 to 10
Mean (SD)	9	5	11	6	8	4	11	6	10	5	8	4
Country of first author												
China	96	31%	8	16%	88	34%	15	25%	17	44%	64	31%
USA	61	20%	13	27%	48	19%	13	22%	5	13%	43	21%
Japan	17	6%	1	2%	16	6%	2	3%	2	5%	13	6%
Germany	14	5%	4	8%	10	4%	4	7%	2	5%	8	4%
Italy	10	3%	3	6%	7	3%	4	7%	0	0%	6	3%
Canada	9	3%	4	8%	5	2%	4	7%	1	3%	4	2%
Spain	8	3%	3	6%	5	2%	3	5%	0	0%	5	2%
United Kingdom	8	3%	2	4%	6	2%	2	3%	2	5%	4	2%
India	7	2%	1	2%	6	2%	1	2%	0	0%	6	3%
Poland	7	2%	2	4%	5	2%	2	3%	0	0%	5	2%
South Korea	7	2%	1	2%	6	2%	1	2%	2	5%	4	2%
Netherlands	5	2%	0	0%	5	2%	0	0%	0	0%	5	2%
Taiwan	5	2%	1	2%	4	2%	1	2%	1	3%	3	1%
Australia	4	1%	0	0%	4	2%	0	0%	0	0%	4	2%
France	4	1%	2	4%	2	1%	2	3%	1	3%	1	0%
Other country	44	14%	4	8%	40	16%	5	8%	6	15%	33	16%
Location of first author												
North America	72	24%	17	35%	55	21%	17	29%	6	15%	49	24%
Asia	139	45%	14	29%	125	49%	22	37%	22	56%	95	46%
Europe	72	24%	17	35%	55	21%	19	32%	10	26%	43	21%
North Africa/Middle East	11	4%	0	0%	11	4%	0	0%	1	3%	10	5%
Oceania	5	2%	0	0%	5	2%	0	0%	0	0%	5	2%
South America	2	1%	0	0%	2	1%	0	0%	0	0%	2	1%
Central America/Caribbean	2	1%	0	0%	2	1%	0	0%	0	0%	2	1%
Sub-Saharan Africa	3	1%	1	2%	2	1%	1	2%	0	0%	2	1%

Additional File 1: Table 1. Characteristics of included studies by sharing type (N=306).

	All articles (N=306)		Actual data sharing				Data sharing					
			Data publicly available (N=49)		Not available (N=257)		Data publicly available (N=59)		Available on request (N=39)		Not available (N=208)	
	N	(%)	N	(%)	N	(%)	N	(%)	N	(%)	N	(%)
2018 Journal Impact Factor												
Median (IQR)	3.3	2.2 to 5.1	4.5	2.8 to 6.7	3.2	2.1 to 4.7	4.3	2.8 to 6.7	3.1	2.6 to 4.8	3.2	2.0 to 4.7
No impact factor	27	9%	4	8%	23	9%	6	10%	2	5%	19	9%
0-5	200	65%	22	45%	178	69%	27	46%	28	72%	145	70%
5-10	63	21%	16	33%	47	18%	18	31%	7	18%	38	18%
10+	16	5%	7	14%	9	4%	8	14%	2	5%	6	3%
Open access												
Closed	155	51%	17	35%	138	54%	19	32%	12	31%	124	60%
Green	14	5%	6	12%	8	3%	6	10%	0	0%	8	4%
Hybrid	21	7%	3	6%	18	7%	4	7%	4	10%	13	6%
Bronze	21	7%	3	6%	18	7%	3	5%	2	5%	16	8%
Gold	95	31%	20	41%	75	29%	27	46%	21	54%	47	23%
Open access												
Yes	151	49%	32	65%	119	46%	40	68%	27	69%	84	40%
No	155	51%	17	35%	138	54%	19	32%	12	31%	124	60%
Citations accrued (Year 1)												
Mean (SD)	3	7	7	15	3	4	7	15	3	4	2	3
Median (IQR)	2	0 to 4	3	1 to 5	2	0 to 3	3	1 to 6	2	1 to 4	2	0 to 3
Citations accrued (Year 2)												
Mean (SD)	6	13	13	26	5	8	13	25	7	7	4	6
Median (IQR)	3	1 to 7	6	3 to 11	3	1 to 6	6	3 to 12	4	1 to 12	3	1 to 5
Journal data sharing policy												
No policy	67	22%	6	12%	61	24%	7	12%	3	8%	57	27%
Encourage	147	48%	14	29%	133	52%	18	31%	22	56%	107	51%
Share on request	4	1%	0	0%	4	2%	0	0%	1	3%	3	1%
Some mandatory	59	19%	15	31%	44	17%	20	34%	11	28%	28	13%
All mandatory	29	9%	14	29%	15	6%	14	24%	2	5%	13	6%
Journal DAS policy												
No policy	239	78%	31	63%	208	81%	35	59%	17	44%	187	90%
Required for trials only	18	6%	4	8%	14	5%	4	7%	1	3%	13	6%
Required for all articles	49	16%	14	29%	35	14%	20	34%	21	54%	8	4%
Journal code sharing policy												
No policy	202	66%	24	49%	178	69%	31	53%	29	74%	142	68%
Encourage	88	29%	15	31%	73	28%	18	31%	7	18%	63	30%
Share on request	6	2%	4	8%	2	1%	4	7%	0	0%	2	1%
Some mandatory	3	1%	0	0%	3	1%	0	0%	2	5%	1	0%
All mandatory	7	2%	6	12%	1	0%	6	10%	1	3%	0	0%
Research area (CSO classification)*												
Biology	69	23%	22	45%	47	18%	26	44%	9	23%	34	16%
Aetiology	20	7%	6	12%	14	5%	6	10%	2	5%	12	6%
Prevention	4	1%	1	2%	3	1%	1	2%	0	0%	3	1%
Detection, diagnosis & prognosis	92	30%	16	33%	76	30%	18	31%	11	28%	63	30%
Treatment	112	37%	14	29%	98	38%	20	34%	20	51%	72	35%
Control, survivorship & outcomes	51	17%	2	4%	49	19%	2	3%	6	15%	43	21%
Study population*												
Human subjects	177	48%	24	49%	153	60%	27	46%	21	54%	129	62%
Human cells	111	36%	26	53%	85	33%	29	49%	20	51%	62	30%
Animal analogues	66	22%	15	31%	51	20%	21	36%	10	26%	35	17%
Animal cells	65	21%	15	31%	50	19%	21	36%	10	26%	34	16%
Commercial cell lines	116	38%	21	43%	95	37%	29	49%	16	41%	71	34%
Other (e.g. simulated data, bacteria)	3	10%	0	0%	3	1%	0	0%	1	3%	2	1%
Collected data from human participants												
Yes	225	74%	38	78%	187	73%	43	73%	29	74%	153	74%
No	81	26%	11	22%	70	27%	16	27%	10	26%	55	26%

*Percentages do not add up to 100% due to multiple answers being possible

Additional File 1: Table 1. Characteristics of included studies by sharing type (N=306).

	All articles (N=306)		Actual data sharing				Data sharing					
			Data publicly available (N=49)		Not available (N=257)		Data publicly available (N=59)		Available on request (N=39)		Not available (N=208)	
	N	(%)	N	(%)	N	(%)	N	(%)	N	(%)	N	(%)
Cancer studied												
Breast cancer	33	11%	6	12%	27	11%	7	12%	4	10%	22	11%
Bowel cancer	25	8%	3	6%	22	9%	3	5%	4	10%	18	9%
Lung cancer	24	8%	5	10%	19	7%	8	14%	4	10%	12	6%
Brain cancer	23	8%	6	12%	17	7%	6	10%	3	8%	14	7%
Liver cancer	22	7%	3	6%	19	7%	4	7%	4	10%	14	7%
Leukaemia	14	5%	7	14%	7	3%	7	12%	1	3%	6	3%
Prostate cancer	11	4%	1	2%	10	4%	1	2%	0	0%	10	5%
Stomach and oesophageal cancer	10	3%	1	2%	9	4%	1	2%	3	8%	6	3%
Head and neck cancer	9	3%	1	2%	8	3%	1	2%	1	3%	7	3%
Lymphoma	9	3%	2	4%	7	3%	3	5%	1	3%	5	2%
Pancreatic cancer	9	3%	1	2%	8	3%	1	2%	2	5%	6	3%
Cholangiocarcinoma	7	2%	1	2%	6	2%	1	2%	2	5%	4	2%
Thyroid cancer	7	2%	2	4%	5	2%	2	3%	1	3%	4	2%
Multiple cancers	48	16%	6	12%	42	16%	7	12%	3	8%	38	18%
Other	55	18%	4	8%	51	20%	7	12%	6	15%	42	20%
Cancer rarity												
Common	150	49%	18	37%	132	51%	24	41%	19	49%	107	51%
Mixed	28	9%	5	10%	23	9%	6	10%	2	5%	20	10%
Rare	118	39%	26	53%	92	36%	28	47%	17	44%	73	35%
Other	10	3%	0	0%	10	4%	1	2%	1	3%	8	4%
Clinical trial												
Yes (Phase III)	4	1%	1	2%	3	1%	1	2%	1	3%	2	1%
Yes (Other phase)	11	4%	0	0%	11	4%	1	2%	1	3%	9	4%
No	291	95%	48	98%	243	95%	57	97%	37	95%	197	95%
Inferential statistics used												
Yes	274	90%	45	92%	229	89%	54	92%	36	92%	184	88%
No	32	10%	4	8%	28	11%	5	8%	3	8%	24	12%
Sample size calculation performed												
Yes	19	6%	4	8%	15	6%	5	8%	5	13%	9	4%
No	255	83%	41	84%	214	83%	49	83%	31	79%	175	84%
Not applicable	32	10%	4	8%	28	11%	5	8%	3	8%	24	12%
Used publicly available data												
Yes (Exclusively)	31	10%	6	12%	25	10%	7	12%	2	5%	22	11%
Yes (Partially)	57	19%	22	45%	35	14%	24	41%	8	21%	25	12%
No	218	71%	21	43%	197	77%	28	47%	29	74%	161	77%
Subjected to a mandatory DAS policy												
Yes	51	17%	14	29%	37	14%	20	34%	22	56%	9	4%
No	255	83%	35	71%	220	86%	39	66%	17	44%	199	96%
Conformed with mandatory DAS policy												
Yes	45	88%	13	93%	32	86%	19	95%	4	44%	22	100%
No	6	12%	1	7%	5	14%	1	5%	5	56%	0	0%
Availability statement present												
Data only	58	19%	11	22%	47	18%	20	34%	33	85%	5	2%
Code only	1	0%	0	0%	1	0%	0	0%	0	0%	1	0%
Data and code	7	2%	7	14%	0	0%	7	12%	0	0%	0	0%
No statement	240	78%	31	63%	209	81%	32	54%	6	15%	202	97%
Statistical analysis software used*												
SPSS	92	30%	8	16%	84	33%	13	22%	13	33%	66	32%
GraphPad	58	19%	15	31%	43	17%	17	29%	8	21%	33	16%
R	37	12%	15	31%	22	9%	16	27%	2	5%	19	9%
SAS	19	6%	3	6%	16	6%	3	5%	3	8%	13	6%
Stata	12	4%	1	2%	11	4%	1	2%	1	3%	10	5%
JMP	5	2%	1	2%	4	2%	1	2%	0	0%	4	2%
MedCalc	5	2%	1	2%	4	2%	1	2%	0	0%	4	2%
Microsoft Excel	5	2%	1	2%	4	2%	1	2%	1	3%	3	1%
Python	4	1%	2	4%	2	1%	2	3%	0	0%	2	1%
Other software	12	4%	4	8%	8	3%	4	7%	0	0%	8	4%
Not reported	70	23%	9	18%	61	24%	8	14%	10	26%	55	26%
Not applicable	32	10%	4	8%	28	11%	5	8%	3	8%	24	12%

*Percentages do not add up to 100% due to multiple answers being possible
